# Clinical and preclinical evidence of meningeal immunity and glymphatic pathways in stroke: a systematic review

**DOI:** 10.3389/fimmu.2026.1885846

**Published:** 2026-07-08

**Authors:** Yuyin Han, Boran Dong, Shuhao Mei, Hailian Yi, Meiting Li, Mengyan Sun, Wenzhen Zhuo, Han Wang, Yong Liu, Xiaoyang Gong

**Affiliations:** 1 Department of Rehabilitation Medicine, The First Affiliated Hospital of Dalian Medical University, Dalian, China; 2 College of Health-Preservation and Wellness, Dalian Medical University, Dalian, China

**Keywords:** AQP4, glymphatic pathway, meningeal immunity, meningeal lymphatic vessels, stroke

## Abstract

Glymphatic transport, meningeal lymphatic drainage, and meningeal immunity have been implicated in metabolic waste clearance, cerebral edema formation, and the regulation of neuroinflammation after stroke. However, the available evidence remains fragmented and has not been systematically integrated. This review was conducted in accordance with PRISMA guidelines. PubMed, Embase, Scopus, the Cochrane Library, and Web of Science were searched from database inception to April 3, 2026, for studies investigating glymphatic pathways, the meningeal lymphatic system, meningeal immune responses, and related imaging biomarkers in patients with clinical stroke or in experimental stroke models. Two investigators independently screened studies, extracted data, and assessed study quality. Because of substantial heterogeneity in study design, imaging methodology, experimental models, and outcome measures, findings were synthesized narratively. A total of 64 studies were included, comprising 17 clinical studies and 47 preclinical studies. Clinical studies, which were largely based on MRI-derived glymphatic imaging biomarkers, suggest that glymphatic dysfunction is associated with stroke severity, cognitive impairment, and clinical outcomes. Preclinical evidence indicates that AQP4 dysregulation, impaired meningeal lymphatic drainage, and border-associated immune activation may influence waste clearance, cerebral edema, inflammation, and tissue repair after stroke. Future studies should establish standardized imaging frameworks, conduct prospective longitudinal validation, define mechanistic differences across stroke subtypes, and facilitate the translation of targeted interventions.

**Systematic Review Registration:**

https://www.crd.york.ac.uk/PROSPERO/home, identifier CRD420261359988.

## 1 Introduction

Stroke is one of the leading neurological causes of death and long-term disability worldwide, posing a major public health challenge (1, 2). Although advances in acute reperfusion therapy, blood pressure management, and comprehensive supportive care have improved outcomes in selected patients, secondary inflammation, cerebral edema, and impaired intracerebral clearance remain closely associated with poor prognosis after stroke (3). In recent years, growing evidence from studies of central nervous system immunity and brain fluid circulation has identified the meningeal immune system as a critical interface between central and peripheral immune responses. In parallel, the glymphatic pathway has been implicated in cerebrospinal fluid–interstitial fluid exchange and metabolic waste clearance. Together, these systems may contribute to post-stroke inflammatory regulation and the maintenance of intracerebral homeostasis (4–6).

Meningeal immunity refers primarily to immune cell populations located within the meninges and associated border structures, together with their regulatory functions. These populations include meningeal macrophages, central nervous system border-associated macrophages, and meningeal lymphatic vessel–associated lymphocytes (7, 8). These cells can clear metabolic waste and inflammatory products from the cerebrospinal fluid. They also regulate immune cell recruitment and inflammatory responses through signaling axes such as CCL2–CCR2, thereby exerting context-dependent and bidirectional effects in pathological conditions such as ischemic stroke and intracerebral hemorrhage (9, 10). The meninges connect meningeal lymphatic drainage with AQP4-dependent glymphatic transport. Immune remodeling within this compartment may influence cerebrospinal fluid–interstitial fluid exchange and waste clearance while propagating border-associated immune responses into the brain parenchyma. Disruption of this pathway after stroke may aggravate cerebral edema, tissue swelling, and waste accumulation (7, 11, 12). Clinical studies have commonly used MRI-visible enlarged perivascular spaces as indirect markers of impaired glymphatic function. These studies have reported associations with early cognitive impairment and unfavorable functional outcomes after ischemic stroke, as well as glymphatic clearance dysfunction in hemorrhagic stroke (13, 14). Accordingly, meningeal immunity and the glymphatic pathway are not only involved in maintaining brain homeostasis but may also play central roles in post-stroke inflammatory amplification, cerebral edema formation, and cognitive impairment (15). Although preclinical and clinical evidence in this field continues to expand (6, 16, 17), existing studies remain highly heterogeneous with respect to stroke subtype, experimental model, observation window, assessment strategy, and endpoint selection (18). A systematic framework integrating the coupled mechanisms of glymphatic transport, meningeal lymphatic drainage, and meningeal immune responses is still lacking. Critical questions remain regarding how glymphatic pathways, meningeal lymphatic drainage, meningeal immune responses, and related imaging or interventional biomarkers are associated with cerebral edema, metabolic waste clearance, neuroinflammation, meningeal lymphatic dysfunction, and neurological or cognitive outcomes in patients with stroke or in experimental stroke models. These associations require evaluation against appropriate comparators, including healthy controls, sham-operated models, contralateral or unaffected brain regions, baseline states, and different stroke subtypes or disease stages. Moreover, it remains necessary to determine how these systems mechanistically contribute to these pathological processes. Accordingly, this review includes eligible clinical and preclinical studies and follows the PRISMA 2020 reporting framework (19). We provide a mechanism-oriented qualitative synthesis and critical appraisal from both clinical and preclinical perspectives. Clinical studies establish patient-level relevance by examining associations between glymphatic-related imaging biomarkers and stroke severity, cognitive impairment, motor dysfunction, and prognosis. In contrast, preclinical studies clarify underlying mechanisms and therapeutic windows by directly interrogating AQP4 polarization, meningeal lymphatic drainage, CSF–interstitial fluid exchange, and border-associated immune responses. Together, clinical observations identify meaningful phenotypes and outcomes, whereas experimental models define their biological basis. This bidirectional integration strengthens mechanistic interpretation, reduces overreliance on indirect imaging biomarkers, and enhances translational value. Collectively, these pathways may act synergistically in post-stroke secondary brain injury and provide a theoretical basis for immune modulation and therapeutic strategies aimed at restoring brain-fluid clearance.

## 2 Methods

This systematic review was conducted in accordance with established methodological guidance for systematic reviews and included a qualitative synthesis of eligible studies. The review protocol was prospectively registered with PROSPERO on April 4, 2026 (registration number: CRD420261359988).

### 2.1 Literature search strategy

The literature search, study screening, and reporting were conducted in accordance with the PRISMA 2020 (19) statement to ensure transparency and methodological rigor in the description of data sources, screening procedures, and inclusion and exclusion criteria. Basic and clinical evidence on meningeal immunity and glymphatic pathways in stroke was systematically summarized through qualitative synthesis and critical appraisal.

A comprehensive search was conducted in PubMed, Embase, Scopus, the Cochrane Library, and Web of Science from database inception to April 3, 2026. The search strategy combined controlled vocabulary terms and free-text terms and covered the core concepts of stroke, meningeal lymphatics, and the glymphatic system. To enhance sensitivity and minimize terminology-related omissions, this review included studies addressing meningeal immune or glymphatic mechanisms and outcomes related to secondary injury after stroke. The aim was to integrate the available evidence and identify translational gaps. The detailed search strategy is presented in **Table 1**.

**Table 1:** Database search strategy.

Search number	Search details
#1	“Stroke”[MeSH Terms]
#2	(((((((((((((((Stroke*[Title/Abstract]) OR (“Cerebrovascular Accident*”[Title/Abstract])) OR (“Cerebral Stroke*”[Title/Abstract])) OR (Stroke*, Cerebral[Title/Abstract])) OR (“Cerebrovascular Apoplexy”[Title/Abstract])) OR (Apoplexy, Cerebrovascular[Title/Abstract])) OR (“Vascular Accident*”, Brain[Title/Abstract])) OR (“Brain Vascular Accident*”[Title/Abstract])) OR (“Cerebrovascular Stroke*[Title/Abstract])) OR (Stroke*, Cerebrovascular[Title/Abstract])) OR (Apoplexy[Title/Abstract])) OR (CVA* (Cerebrovascular Accident[Title/Abstract]))) OR (Stroke*, Acute[Title/Abstract])) OR (“Acute Stroke*”[Title/Abstract])) OR (“Cerebrovascular Accident*”, Acute[Title/Abstract])) OR (“Acute Cerebrovascular Accident*”[Title/Abstract])
#3	#1 OR #2
#4	“Glymphatic System”[MeSH Terms]
#5	“Glymphatic System”[Title/Abstract] OR “Glymphatic Clearance System”[Title/Abstract] OR “glymphatic pathway*”[Title/Abstract] OR ((“pathway”[All Fields] OR “pathway s”[All Fields] OR “pathways”[All Fields]) AND “Glymphatic”[Title/Abstract]) OR “meningeal lymphatic vessel*”[Title/Abstract] OR ((“lymphatic vessels”[MeSH Terms] OR (“lymphatic”[All Fields] AND “vessels”[All Fields]) OR “lymphatic vessels”[All Fields] OR (“lymphatic”[All Fields] AND “vessel”[All Fields]) OR “lymphatic vessel”[All Fields]) AND “Meningeal”[Title/Abstract]) OR “virchow robin space*”[Title/Abstract] OR “space virchow robin”[Title/Abstract] OR “Virchow Robin Space”[Title/Abstract] OR “brain perivascular space*”[Title/Abstract] OR (“Perivascular”[All Fields] AND “space brain”[Title/Abstract])
#6	#4 OR #5
#7	#3 AND #6

In this study, the search strategy of the PubMed database was used as an example, and the search strategy of other databases was formulated according to the search method preset in the registration scheme.

### 2.2 Inclusion and exclusion criteria

#### 2.2.1 Inclusion criteria

This study used a structured inclusion framework applicable to both clinical and preclinical studies, combined with mechanism-based relevance criteria. The aim was to integrate evidence on post-stroke meningeal immunity, meningeal lymphatic drainage, and glymphatic pathways, rather than to quantitatively evaluate the effects of specific interventions. The specific inclusion criteria were as follows (1): Clinical studies were required to include patients with non-traumatic stroke confirmed by clinical diagnosis or neuroimaging, including ischemic stroke, recent small subcortical infarction, spontaneous intracerebral hemorrhage, and non-traumatic subarachnoid hemorrhage. Studies examining post-stroke cognition, motor function, mood, fatigue, cerebral edema, or prognosis were included only when the study population had a clearly defined stroke event. Healthy or non-stroke participants were eligible only as control groups; (2) Preclinical studies were required to use animal models that explicitly simulated non-traumatic ischemic or hemorrhagic stroke. Studies using mixed disease models were included only when stroke-related data could be extracted separately; (3) Studies were required to directly assess post-stroke meningeal immunity, meningeal lymphatic drainage, or glymphatic pathways, including border-associated immune responses, AQP4 expression or polarization, CSF–ISF exchange, deep cervical lymph node drainage, or related MRI or functional imaging metrics; (4) Studies were required to report at least one clinical, imaging, or mechanistic outcome. Only peer-reviewed, full-text original research articles published in English were included, comprising clinical observational studies, quantitative imaging studies, clinical intervention studies, and animal or mechanistic studies of stroke.

#### 2.2.2 Exclusion criteria

To ensure the focus and reliability of the analysis, studies meeting any of the following criteria were excluded: (1) Studies without a clearly defined recent clinical or neuroimaging-confirmed stroke event, including those involving only vascular risk factors or asymptomatic imaging burden of cerebral small vessel disease; (2) Studies of traumatic brain injury, traumatic cerebrovascular injury, traumatic intracranial hemorrhage or subarachnoid hemorrhage, and iatrogenic, surgical, or other non-spontaneous vascular injuries; (3) Studies involving isolated chronic cerebral hypoperfusion or vascular cognitive impairment models. Studies were also excluded if they combined these conditions with acute focal ischemic or hemorrhagic stroke but did not allow separate extraction of stroke-related data; (4) Studies of non-stroke neurological diseases or models, or studies involving immunity, lymphatics, pharmacological exposure, or oxygen exposure that did not directly assess post-stroke meningeal immunity, meningeal lymphatic drainage, or glymphatic function; (5) Reviews, case reports, conference abstracts, editorials, preprints, non-peer-reviewed publications, studies with incomplete data, studies for which the full text was unavailable, and duplicate publications.

### 2.3 Study selection process

**Figure 1:**
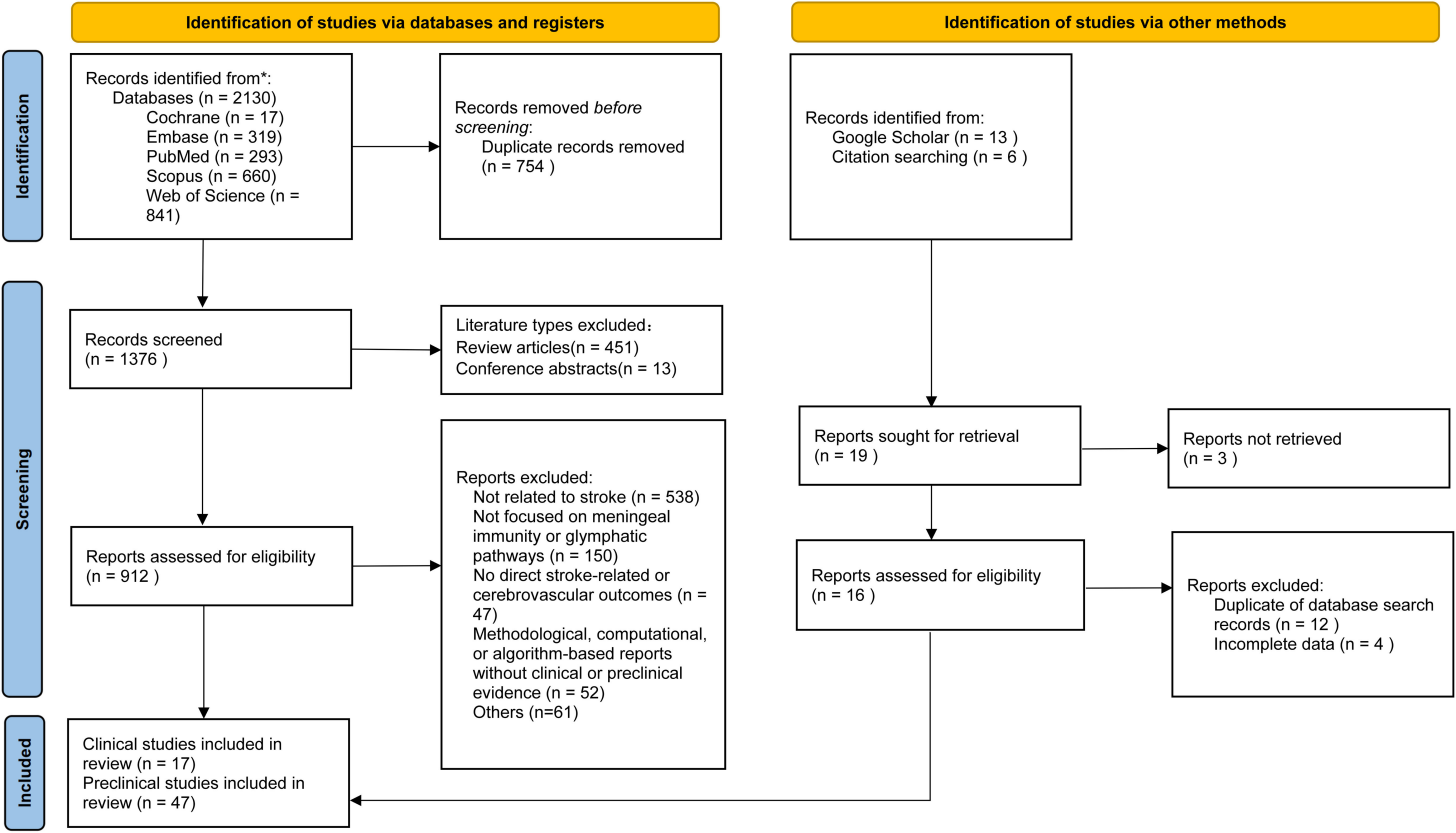
Flow diagram of literature identification and screening.

Study selection was reported in accordance with the PRISMA 2020 flow diagram. After duplicate records were removed, two investigators independently screened titles and abstracts and assessed full-text articles against the eligibility criteria. Disagreements were resolved through discussion or consultation with a third investigator. A total of 2,130 records were identified through the literature search. Following a standardized screening workflow, 64 studies were ultimately included as the evidence base for this systematic review. The detailed screening process is shown in **Figure 1**. In accordance with the Preferred Reporting Items for Systematic Reviews and Meta-Analyses (PRISMA) 2020 statement, the identification, screening, and inclusion process for this review are presented in **Figure 1**.

### 2.4 Data extraction and qualitative synthesis

Systematic data extraction was performed for all 64 studies included in this review. Extracted data included model or species, observation window, primary assessment methods, interventions, and outcomes. These variables were subsequently analyzed through qualitative synthesis.

### 2.5 Literature quality assessment

Given the methodological diversity of the included studies, quality assessment was performed using design-specific appraisal tools. Preclinical animal studies were evaluated using the Systematic Review Center for Laboratory Animal Experimentation (SYRCLE) risk of bias tool. For clinical studies, cross-sectional studies were assessed using the Joanna Briggs Institute (JBI) Critical Appraisal Checklist for Analytical Cross-Sectional Studies, cohort studies were evaluated using the Newcastle–Ottawa Scale (NOS), and the included prospective randomized interventional study was assessed using the Cochrane Risk of Bias 2 (RoB 2) tool. Because of substantial heterogeneity across the included studies in terms of study populations, stroke subtypes, outcome measures, assessment methods, and study designs, quantitative synthesis was not appropriate; therefore, only a qualitative synthesis was performed.

## 3 Overview of evidence on meningeal immunity and glymphatic pathways in stroke

This systematic review included 64 studies, comprising 47 preclinical animal studies and 17 clinical investigations. The evidence is predominantly derived from mechanistic preclinical research, with clinical imaging studies providing complementary validation. Preclinical studies employed diverse stroke models and focused on glymphatic function, aquaporin-4 (AQP4), meningeal lymphatics, border-associated immune cells, and the clearance of pathological substrates. Collectively, these studies indicate that these mechanisms contribute to cerebral edema, neuroinflammation, and impaired functional recovery. Among the clinical studies, 6 were cross-sectional, 11 were cohort studies, and 1 was a prospective randomized interventional trial. Clinical investigations generally exhibited a relatively high risk of bias, with most domains rated as unclear in the SYRCLE assessment. In contrast, preclinical studies were largely published in recent high-impact journals. The methodological quality of clinical studies ranged from acceptable to high: cross-sectional studies assessed by the JBI tool were rated acceptable to good; cohort studies scored 6–9 on the Newcastle-Ottawa Scale (NOS); and the single RoB-2–assessed interventional study showed concern in one high-risk domain. Stratified by study type, preclinical research accounted for 72.3% of included studies and primarily aimed to elucidate mechanistic pathways and identify potential therapeutic targets. Clinical studies accounted for 27.7% and comprised cross-sectional, cohort, and a small number of interventional studies. These studies primarily employed imaging-based metrics—including the DTI-ALPS index, enlarged perivascular spaces, choroid plexus volume, free-water imaging, and functional imaging—to assess the relationship between glymphatic function and post-stroke outcomes, such as cognitive impairment, motor deficits, cerebral edema, depression, fatigue, and overall prognosis. Collectively, current evidence supports the concept that meningeal immunity, the glymphatic system, and meningeal lymphatic drainage are critical intersecting mechanisms underlying secondary injury and functional recovery after stroke. However, substantial heterogeneity in study design, stroke models, assessment methods, and outcome measures necessitates a descriptive synthesis stratified by study type, stroke subtype, and pathway category (**Tables 2**, **3**).

**Table 2:** Quality assessment of included clinical studies.

Analytical cross-sectional studies: Joanna Briggs Institute(JBI)										
Number	Study	Q1	Q2	Q3	Q4	Q5	Q6	Q7	Q8	
1	Tu et al., 2024 (20)	Y	Y	Y	Y	Y	Y	Y	Y	
2	Qin et al., 2023 (21)	Y	U	Y	U	U	U	Y	Y	
3	Toh et al., 2021 (17)	Y	Y	Y	Y	Y	Y	Y	Y	
4	Zeng et al., 2025 (22)	Y	Y	Y	Y	Y	U	Y	Y	
5	Sun et al., 2025 (23)	Y	Y	Y	Y	U	N	Y	Y	
6	Huang et al., 2025 (24)	Y	Y	Y	Y	Y	Y	Y	Y	
Cohort studies: newcastle–ottawa scale (NOS)										
Number	Study	Selection				Comparability	Exposure			Total
		Q1	Q2	Q3	Q4		Q1	Q2	Q3	
1	Kim et al., 2022 (25)	1	0	1	1	1	1	1	0	6
2	Chen et al., 2025 (26)	1	1	1	1	1	1	1	0	7
3	Lin et al., 2025 (27)	1	0	1	1	1	1	1	0	6
4	Qiu et al., 2025 (28)	1	0	1	1	1	1	1	0	6
5	Zhang et al., 2025 (73)	1	1	1	1	1	1	1	0	7
6	Wang et al., 2025 (29)	1	1	1	1	1	1	1	0	7
7	Chao et al., 2024 (30)	1	1	1	1	1	1	1	0	7
8	Bian et al., 2025 (15)	1	1	1	1	1	1	1	0	7
9	Alvim et al., 2026 (31)	1	1	1	1	2	1	1	1	9
10	Qu et al., 2026 (32)	1	1	1	1	2	1	1	1	9
Prospective randomized interventional study: cochrane risk of bias 2 (RoB-2) tool										
Number	Study	Q1	Q2	Q3	Q4	Q5			Q6	Q7
1	Dong et al., 2026 (33)	S	S	L	S	H			S	S

**Table 3:** Quality assessment of preclinical studies.

Animal studies: systematic review centre for laboratory animal experimentation (SYRCLE)											
Number	Study	Q1	Q2	Q3	Q4	Q5	Q6	Q7	Q8	Q9	Q10
1	Sun et al., 2022 (34)	Y	U	U	U	U	U	U	N	Y	U
2	Zhang et al., 2026 (12)	Y	U	U	U	U	U	U	U	Y	U
3	Li et al., 2026 (35)	U	U	U	U	U	U	U	N	Y	U
4	Yang et al., 2024 (44)	Y	U	U	U	Y	U	U	U	Y	U
5	Mestre et al., 2020 (37)	U	U	U	U	U	U	U	U	Y	U
6	Yu et al., 2026 (38)	Y	U	U	U	U	U	Y	U	Y	U
7	Pedragosa et al., 2018 (39)	Y	U	Y	U	Y	U	Y	N	Y	U
8	Bai et al., 2022 (40)	U	U	U	U	U	U	Y	N	Y	U
9	Tian et al., 2025 (41)	Y	U	U	U	U	U	U	N	Y	U
10	Rajan et al., 2020 (42)	U	U	U	U	U	U	U	U	Y	U
11	Lyu et al., 2021 (43)	Y	U	U	U	U	U	U	U	Y	U
12	Yang et al., 2024 (36)	U	U	U	U	U	U	U	U	Y	U
13	Yang et al., 2023 (45)	Y	U	U	U	U	U	Y	U	Y	U
14	Howe et al., 2018 (46)	Y	U	U	U	Y	U	Y	U	Y	U
15	Tsai et al., 2022 (47)	Y	U	U	U	U	U	Y	N	Y	U
16	Zhu et al., 2024 (48)	Y	U	U	U	U	U	U	N	Y	U
17	Li et al., 2022 (49)	Y	U	U	U	U	U	U	U	Y	U
18	Kim et al., 2025 (50)	U	U	U	U	U	U	U	U	Y	U
19	Keuters et al., 2025 (51)	U	U	U	U	U	U	U	U	Y	U
20	Gaberel et al., 2014 (52)	U	U	U	U	U	U	U	U	Y	U
21	Sun et al., 2025 (76)	Y	U	U	U	U	U	Y	U	Y	U
22	Lin et al., 2020 (53)	Y	U	U	U	U	U	U	U	Y	U
23	Gu et al., 2025 (54)	Y	U	U	U	U	U	U	N	Y	U
24	Zhang et al., 2022 (74)	Y	U	U	U	Y	U	U	U	Y	U
25	Yanev et al., 2020 (56)	Y	U	U	U	Y	U	U	N	Y	U
26	Riew et al., 2022 (57)	U	U	U	U	Y	U	Y	U	Y	U
27	Arbel-Ornath et al., 2013 (58)	U	U	U	U	U	U	U	U	Y	U
28	Li et al., 2023 (59)	U	U	U	U	U	U	U	U	Y	U
29	Yi et al., 2022 (60)	U	U	U	U	U	U	U	U	Y	U
30	Chen et al., 2025 (26)	U	U	U	U	U	U	U	N	Y	U
31	Cai et al., 2026 (78)	Y	U	U	U	Y	U	Y	N	Y	U
32	Wang et al., 2026 (10)	U	U	U	U	U	U	U	U	Y	U
33	Li et al., 2025 (13)	Y	U	U	U	Y	U	Y	U	Y	U
34	Li et al., 2023 (79)	U	U	U	U	U	U	U	U	Y	U
35	Hsiao et al., 2025 (61)	Y	U	U	U	U	U	U	U	Y	U
36	Liu et al., 2023 (62)	U	Y	U	U	U	U	U	N	Y	U
37	Wang et al., 2023 (63)	U	U	U	U	U	U	U	U	Y	U
38	Zhu et al., 2025 (64)	U	U	U	U	U	U	U	N	Y	U
39	Goulay et al., 2017 (65)	U	U	U	U	U	U	U	U	Y	U
40	Li et al., 2025 (66)	Y	U	U	U	U	U	Y	U	Y	U
41	Back et al., 2020 (67)	Y	Y	U	U	Y	U	Y	N	Y	U
42	Li et al., 2024 (68)	Y	U	U	U	U	U	U	U	Y	U
43	Boisserand et al., 2024 (69)	U	U	U	U	U	U	U	U	Y	U
44	Lyu et al., 2021 (70)	Y	U	U	U	U	U	U	N	Y	U
45	Yu et al., 2026 (75)	U	U	U	U	U	U	U	U	Y	U
46	Luo et al., 2024 (71)	U	U	U	U	U	U	U	U	Y	U
47	Yu et al., 2024 (72)	U	U	U	U	U	U	U	U	Y	U

## 4 Clinical Manifestations of Glymphatic-Related Imaging Biomarkers in Stroke

Post-stroke glymphatic dysfunction is not represented by a single imaging abnormality. Rather, it reflects a composite imaging phenotype involving impaired cerebrospinal fluid–interstitial fluid (CSF–ISF) exchange, perivascular fluid retention, and neurovascular dysfunction. At present, the diffusion tensor imaging analysis along the perivascular space (DTI-ALPS) index is the most widely used functional imaging metric. It indirectly reflects water-molecule diffusivity along the direction of the perivascular spaces surrounding the deep medullary veins (**Table 4**). In DTI-ALPS studies, Zhang et al. (73) showed that reduced ALPS after stroke reflects not only restricted water diffusion around the lesion but also a broader reduction in intracerebral clearance efficiency after acute injury. Partial recovery of ALPS on the infarct side during rehabilitation suggests that glymphatic dysfunction is not a static impairment, but may gradually improve as edema resolves, inflammation subsides, and the neurovascular unit undergoes remodeling. The association between early ipsilesional ALPS and 6-month cognitive outcomes extends the clinical relevance of this marker beyond the identification of impaired clearance, supporting its potential value for assessing post-stroke fluid-dynamic recovery trajectories and long-term prognostic risk. Toh and Siow (17) further showed that ALPS values were significantly lower on the lesion side than on the contralateral side in patients with ischemic stroke. ALPS was also associated with time since stroke onset, with higher ALPS values observed at later time points. This finding supports a temporal interpretation of ALPS changes. Early reductions in ALPS may indicate compression or obstruction of local pathways by edema, inflammation, and tissue disruption, whereas subsequent increases may reflect gradual restoration of perivascular fluid movement or a transition of the perilesional diffusion environment from acute disorganization toward relative stabilization. Thus, ALPS may reflect both local impairment of clearance and the recovery trajectory of post-stroke fluid dynamics. However, it should not be regarded as a direct measure of whole-brain glymphatic flow, but rather as an indirect estimate of perivascular diffusion status in specific white-matter regions.

**Table 4:** Clinical studies evaluating glymphatic- and perivascular/meningeal-lymphatic-related imaging markers in stroke.

Study	Population	Stroke type/stage	Design/timing	Domain	Marker(s)	Main comparison	Linked outcome
Ischemic stroke/post-stroke states							
DTI-ALPS/MRI-derived glymphatic markers							
Chen et al., 2025 (26)	51 chronic stroke; 27 HC	Chronic, 3–12 mo	Retrospective; 3 mo & 1 y	ALPS proxy	Lesion/contra ALPS	Stroke vs HC; lesion vs contra	Cognition
Lin et al., 2025 (27)	82 cerebral infarction	Infarction; 90-d outcome	Retrospective	ALPS proxy	L/R/mean ALPS	Good vs poor 90-d mRS	Functional outcome
Qin et al., 2023 (21)	Subacute IS; 32 HC	Subacute IS	Cross-sectional	ALPS proxy	ALPS + CST	IS vs HC; ALPS vs FMA	Motor
Zhang et al., 2025 (73)	51 IS; 95 non-stroke	IS; pre/post rehab	T1 vs T2; 6-mo PSCI	Multimodal GS	ALPS, CPV, PVS	Stroke vs non-stroke; T1 vs T2	PSCI
Wang et al., 2025 (29)	29 subcortical infarct; 25 HC	Acute; 7 & 90 d	Prospective	ALPS proxy	Bilateral ALPS	IS vs HC; ALPS vs MoCA	Early cognition
Toh & Siow, 2021 (17)	50 IS; 44 HC	IS; onset varied	Retrospective	ALPS proxy	ALPS ratios	Lesion vs contra; vs HC	Recovery trend
Zeng et al., 2025 (22)	59 AIS; 29 HC	Acute; 2-mo subset	Prospective	ALPS proxy	ALPS	AIS vs HC; baseline vs follow-up	Upper-limb motor
Chao et al., 2024 (30)	96 stroke; 44 HC	PSD framework	Prospective + follow-up	ALPS + NVC	ALPS; CBF-BOLD NVC	Stroke vs HC; ALPS/NVC vs PSD	Depression
Sun et al., 2025 (23)	PSF vs non-PSF IS	First IS	Retrospective case-control	ALPS + rs-fMRI	ALPS; connectivity	PSF vs non-PSF	Fatigue
Bian et al., 2025 (15)	48 capsular; 28 pontine; 50 HC	<7 d and 90 d	Prospective longitudinal	ALPS + CVR	ALPS; CVR	CS/PS vs HC; CVR-ALPS	Functional reorganization
Alvim et al., 2026 (31)	189 mild IS	Mild IS + SVD	Design not explicit	ALPS/SVD	ALPS, PVS, WMH	ALPS vs SVD & MoCA	Cognition: null MoCA
Dong et al., 2026 (33)	35 IS with arm deficit	Rehab; 2-wk rTMS	Prospective randomized	Interventional ALPS	Bilateral ALPS	HF vs LF rTMS	Motor recovery
Qu et al., 2026 (32)	104 AIS; 3-mo follow-up	Subacute post-AIS	Prospective cohort	Lateralized ALPS	L/R/mean ALPS	Depressive vs non-depressive	Mood
EPVS/PVS/CPV-related studies							
Tu et al., 2024 (20)	176 AIS; 32 controls	Early AIS, ≤1 mo	Cross-sectional	PVS proxy	EPVS; BG-EPVS	CI vs no CI	Early cognition
Qiu et al., 2026 (28)	66 RSSI	3–7 d to 3 y	Prospective longitudinal	Fluid dynamics	FW, PVS, ALPS	Ipsilateral vs contra; longitudinal	Attention
Huang et al., 2025 (24)	32 post-stroke; 27 HC	6 mo post-stroke	Timing stated	Multimodal proxy	ALPS, CPV, PVS	Stroke vs HC; markers vs cognition	Cognition
Hemorrhagic stroke (ICH/SAH)							
DTI-ALPS/MRI-derived glymphatic markers							
Zhang et al., 2022 (55)	20 sICH; 31 HC	Spontaneous ICH	Duration analyzed	ALPS proxy	DTI-ALPS	Lesion vs contra; vs HC	Disease duration
EPVS/PVS/CPV-related studies							
Kim et al., 2022 (25)	139 aSAH; 99 follow-up	aSAH; MRI ≤1 mo	Retrospective follow-up	PVS proxy	BG/CSO ePVS	Baseline vs follow-up	PVS progression

Clinical studies increasingly suggest that stroke is accompanied by disturbances in glymphatic and perivascular fluid clearance. In ischemic stroke, the DTI-ALPS index is commonly reduced, particularly in the lesional hemisphere, and may partially normalize over time or during rehabilitation. Lower ALPS values have been associated with motor deficits, cognitive impairment, fatigue, and depressive symptoms. Other imaging markers, including the burden of PVS/EPVS and increased choroid plexus volume, have also been related to specific cognitive outcomes. In hemorrhagic stroke, reduced lesion-side ALPS values in spontaneous intracerebral hemorrhage and progressive CSO-ePVS after aneurysmal subarachnoid hemorrhage suggest that hemorrhagic injury may compromise brain fluid clearance. Overall, these observations highlight the potential utility of glymphatic dysfunction as an imaging biomarker and clinically relevant indicator in stroke.

On this basis, the clinical relevance of ALPS extends from disease dynamics to prognostic assessment and rehabilitation monitoring. Lin et al. (27) and Dong et al. (33), from the perspectives of natural prognosis and rehabilitation intervention, respectively, reported that ALPS alterations were associated with post-stroke functional outcomes, activities of daily living, and upper-limb motor recovery. The shared value of these studies is that they advance ALPS from a purely imaging marker to a functional indicator of post-stroke repair status. Reduced ALPS may indicate insufficient perivascular clearance capacity, which could facilitate the retention of inflammatory mediators, metabolic waste, and necrotic debris, thereby affecting neural network reconstruction and functional recovery. However, changes in ALPS after rehabilitation do not always show a unidirectional increase, suggesting that ALPS may also be influenced by interhemispheric reorganization, cortical excitability modulation, and local vascular responses. Therefore, ALPS in prognostic and rehabilitation studies should not be interpreted simply as a direct measure of clearance capacity. Instead, its interpretation should integrate lesion laterality, intervention type, scanning time window, and neural network remodeling. Qin (21) and Zeng et al. (22) both found that reduced ipsilesional ALPS was associated with motor function, neurological deficits, or recovery status in patients with ischemic stroke at different disease stages. These findings suggest that reduced ALPS may represent not only an imaging manifestation of impaired local fluid exchange but also a marker related to stroke burden and recovery potential. In hemorrhagic stroke, Zhang et al. (55) reported reduced ipsilesional ALPS in patients with spontaneous intracerebral hemorrhage, with changes related to disease course. This finding suggests that persistent local clearance impairment may exist around the hematoma. Potential mechanisms may involve increased perivascular pathway burden caused by blood degradation products, iron deposition, coagulation components, and secondary inflammation. Compared with ischemic stroke, studies have suggested that ALPS reduction after intracerebral hemorrhage may more prominently reflect insufficient clearance of metabolic waste and inflammatory products around the hematoma, which may contribute to the sustained development of edema, oxidative stress, and glial responses (25, 31). Structural markers, such as enlarged perivascular spaces (EPVS), provide another perspective on glymphatic burden. A follow-up study of subarachnoid hemorrhage showed that EPVS burden may worsen over the disease course and is associated with hemorrhage severity and baseline PVS burden (20). In ischemic stroke, patients with early cognitive impairment often show increased EPVS in the basal ganglia, greater white-matter hyperintensity burden, brain atrophy, and larger lesion volume. Qiu et al. (28) further showed that changes in PVS volume, free water, and ALPS after small subcortical infarction may reflect abnormalities in intracerebral fluid dynamics, although their associations with cognitive trajectories were not stable. Therefore, the clinical interpretation of structural fluid markers should account for lesion location, observation window, and the background burden of cerebral small vessel disease, rather than treating these markers as isolated prognostic indicators. Cognitive impairment is an important direction for the clinical translation of glymphatic imaging. Reduced ALPS after acute subcortical infarction has been associated with cognitive performance in the early phase and at 90 days (29), suggesting that cognitive impairment may evolve from early local clearance dysfunction into a more widespread reduction in whole-brain clearance efficiency. Huang et al. (24) further showed that reduced ALPS and increased choroid plexus volume after stroke were associated with impairments in working memory, processing speed, and executive function. In contrast, the chronic-phase follow-up study by Chen et al. (26) suggested that ALPS remained lower than that in healthy controls over the long term, but showed limited associations with specific cognitive or motor domains. Therefore, ALPS may be more appropriately considered a background marker of long-term clearance impairment and disrupted brain homeostasis after stroke, rather than a direct predictor of impairment in a single cognitive domain.

In recent years, increasing attention has been paid to the relationship between glymphatic imaging abnormalities, post-stroke neuropsychiatric symptoms, and neurovascular dysfunction. Chao et al. (30) found that post-stroke glymphatic abnormalities should not be viewed solely as impaired fluid clearance, but may also represent an important manifestation of neurovascular unit dysfunction. The mediating role of ALPS between abnormal neurovascular coupling and depressive symptoms suggests a potential pathological continuum linking perfusion regulation, neural activity, and metabolic waste clearance. When cerebral blood flow responses do not match neural activity demands after stroke, vascular pulsatility and CSF–ISF exchange efficiency may decline, promoting the retention of inflammatory mediators and metabolic products and thereby affecting emotion-related network function. Thus, post-stroke depression may be associated with both impaired neurovascular coupling and insufficient glymphatic clearance. Sun et al. (23) found that patients with post-stroke fatigue had lower ALPS values across multiple brain regions than patients without fatigue. ALPS was negatively correlated with fatigue severity and was accompanied by abnormal functional connectivity. These findings suggest that post-stroke fatigue may not merely reflect subjective symptoms or psychological responses, but may be related to reduced brain network efficiency, white-matter pathway injury, and insufficient metabolic waste clearance. Clearance impairment, represented by low ALPS, may promote the persistent retention of inflammatory factors, lactate, and other metabolic products, thereby reducing neural network efficiency and manifesting as persistent fatigue and reduced tolerance to recovery. Bian et al. (15) further showed a lesion-location-dependent relationship between cerebrovascular reactivity (CVR) reorganization and reduced ALPS. These findings indicate that glymphatic imaging biomarkers may reflect not only local lesion-related injury but also interactions among perfusion regulation, brain network reorganization, metabolic waste clearance, and neuropsychiatric symptoms. In summary, post-stroke glymphatic-related imaging biomarkers are mainly characterized by impaired functional diffusion, structural fluid retention, and abnormal neurovascular coupling. DTI-ALPS primarily reflects perivascular water diffusivity and potential clearance function, whereas EPVS, free water, and choroid plexus volume more closely reflect fluid retention and structural clearance burden. Metrics derived from arterial spin labeling (ASL), blood oxygen level–dependent (BOLD) imaging, and cerebrovascular reactivity (CVR) assessment may help reveal the relationship between neurovascular regulation and glymphatic function. These markers provide imaging evidence of post-stroke glymphatic abnormalities; however, most remain indirect indicators and are susceptible to the effects of white-matter injury, lesion location, cerebral small vessel disease burden, and scanning parameters. Future studies should establish standardized, multimodal, and longitudinal assessment frameworks and integrate inflammatory markers, meningeal lymphatic function, and long-term clinical outcomes to further define the clinical value of these biomarkers as glymphatic–immune imaging markers and potential interventional targets.

## 5 Preclinical advances in glymphatic dysfunction and meningeal immune responses in ischemic stroke

### 5.1 Glymphatic dysfunction, edema formation, impaired clearance, and secondary injury after ischemic stroke

Clinical imaging studies suggest that glymphatic dysfunction occurs after stroke, whereas preclinical studies further indicate that this abnormality is not merely a disorder of fluid transport. Rather, it represents a continuous pathological process spanning hyperacute edema initiation, impaired clearance from the acute to subacute phases, and secondary injury in remote brain regions. In this context, the glymphatic system may serve as an important hub linking disrupted cerebrospinal fluid (CSF) dynamics, impaired meningeal immune drainage, and sustained neuroinflammation. In the hyperacute phase, glymphatic abnormalities first manifest as abnormal entry of CSF into the ischemic brain parenchyma along perivascular pathways (41). In a middle cerebral artery occlusion (MCAO) model, Mestre et al. (37) found that CSF tracers rapidly entered the ipsilateral hemisphere within minutes after ischemia, accompanied by an early increase in cortical water content. This study shifted the conventional understanding of early post-stroke edema, which had largely emphasized cytotoxic edema and blood–brain barrier disruption, by suggesting that CSF itself may also represent an important fluid source for acute edema. However, early abnormal influx represents only one aspect of glymphatic dysfunction. The pathological evolution after stroke also involves subsequent impairment of clearance capacity and restricted efflux. During the formation and progression of vasogenic edema after ischemia, the clearance component of the glymphatic system is among the first to become functionally impaired. This impairment is characterized by restricted interstitial fluid outflow and reduced drainage efficiency for metabolic waste and inflammation-related molecules. In an ischemia–reperfusion model, Yang et al. (36) observed that edema progression was accompanied by increased intracerebral tracer retention and reduced drainage to the deep cervical lymph nodes, indicating that post-stroke glymphatic dysfunction involves not only abnormal influx but also failure of clearance and drainage. Together with evidence of altered AQP4 polarization and disruption of perivascular structures, these findings suggest that dysfunction of astrocytic endfeet may provide an important structural basis for reduced glymphatic clearance. At this stage, glymphatic dysfunction may exacerbate secondary injury after ischemia by prolonging edema, promoting glial responses, and facilitating the retention of inflammatory mediators. The effects of impaired glymphatic clearance are not limited to the peri-infarct region but may also contribute to secondary neurodegeneration in remote brain areas (53). In a cortical infarction model, reduced glymphatic clearance was observed in the ipsilateral thalamus, a region connected to the primary lesion through fiber pathways. This was accompanied by tracer retention, perivascular Aβ deposition, astroglial activation, and neuronal loss. Inhibition of glymphatic clearance further aggravated injury in remote brain regions, as well as sensory and cognitive deficits. These findings indicate that inefficient glymphatic clearance not only affects edema resolution and inflammatory clearance around the infarct but may also promote the extension of focal ischemic injury toward secondary neurodegeneration in remote brain regions (54, 58, 67). In summary, glymphatic dysfunction after ischemic stroke can be understood as a dynamic continuum. The hyperacute phase is dominated by abnormal CSF influx and the initiation of early edema. The acute to subacute phases are characterized by disrupted AQP4 polarization, reduced fluid efflux, and impaired clearance of metabolic waste. In the later phase, glymphatic dysfunction is associated with abnormal protein deposition, sustained glial responses, and secondary neurodegeneration. Thus, the glymphatic system is involved not only in post-stroke fluid transport but may also influence cerebral edema, inflammation resolution, and long-term neural repair. Future studies should clarify the relative contributions of abnormal influx and insufficient clearance at different stages and evaluate the therapeutic value of targeting AQP4 polarization, perivascular spaces, and meningeal lymphatic efflux (**Table 5**).

**Table 5:** Preclinical evidence linking ischemic stroke–associated meningeal/CSF–ISF transport abnormalities with cerebral edema progression.

Study	Model/species	Time window	Main assessment methods	Direction of evidence
Mestre et al., 2020 (37)	MCAO; mouse	Seconds to 30 min	CSF tracer imaging; MRI; two-photon microscopy	CSF influx in the hyperacute phase↑
Gaberel et al., 2014 (52)	Occlusive ischemic stroke; mouse	Acute phase	Contrast-enhanced MRI; clearance tracer analysis	Impaired solute clearance
Zhang et al., 2022 (74)	pMCAO; mouse	Early acute to subacute phase	BOPTA-Gd–enhanced MRI; ADC mapping; AQP4-related indices	Early inflow↑→Late clearance↓
Zhu et al., 2024 (48)	tMCAO with reperfusion; mouse	Days 1–7	MRI; CSF tracer imaging; dCLN drainage assessment	Recovery of clearance correlates with resolution of cerebral edema

Collectively, these studies suggest that impaired intracerebral fluid homeostasis after ischemic stroke is involved throughout the evolution of brain edema. Early after stroke, enhanced CSF influx and increased parenchymal fluid accumulation predominate, whereas later phases are marked by defective glymphatic/interstitial fluid clearance and impaired lymphatic drainage. These alterations may delay interstitial solute clearance, promote metabolic waste retention, and hinder tissue repair. Thus, post-stroke brain edema should not be viewed solely as a consequence of vascular injury and barrier disruption, but also as a manifestation of sustained imbalance in brain fluid transport and clearance pathways.

### 5.2 AQP4-related mechanisms of glymphatic dysfunction

**Figure 2:**
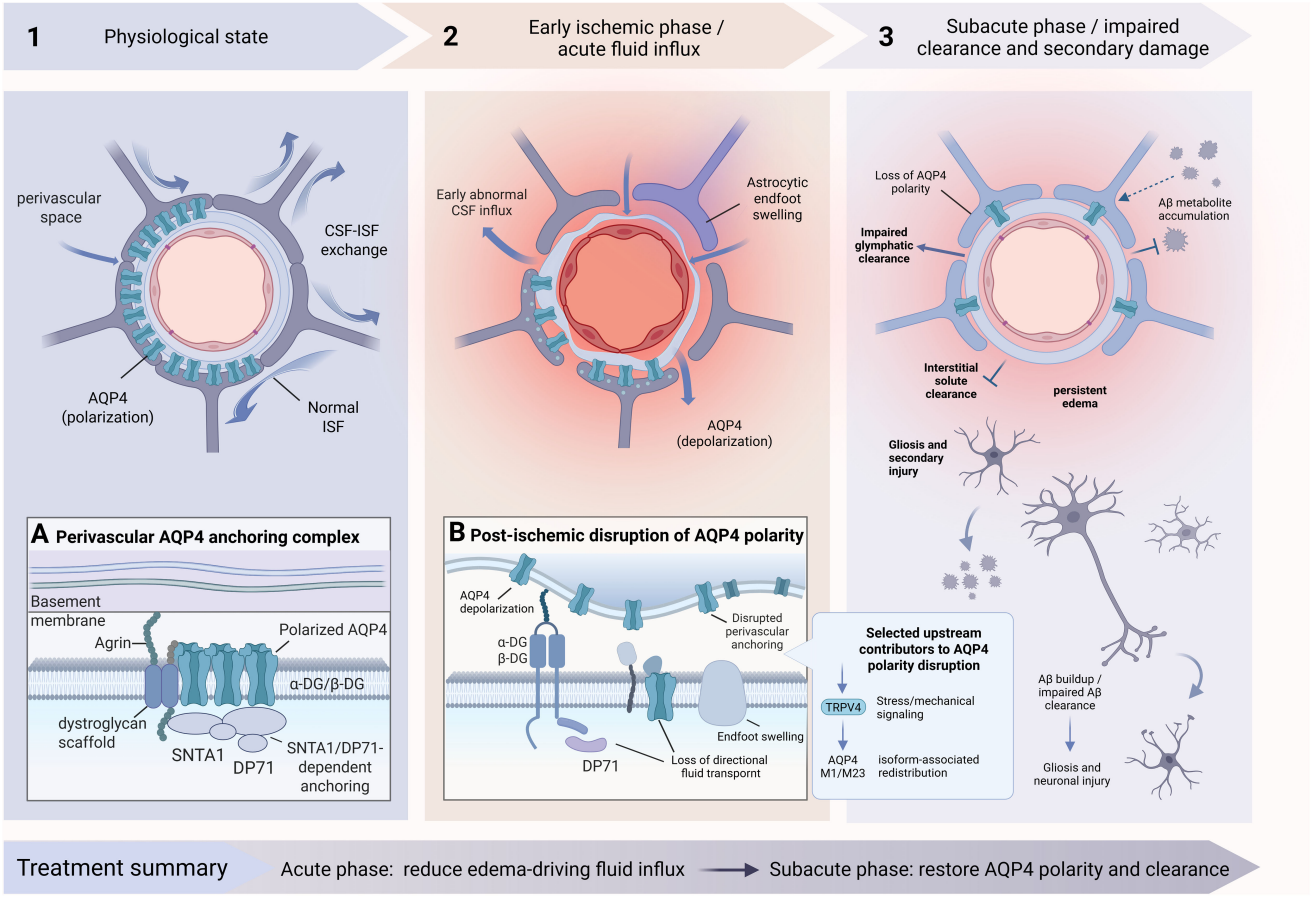
Glymphatic dysfunction after ischemic stroke. After ischemic stroke, disruption of perivascular fluid transport impairs CSF influx, interstitial fluid exchange, and waste clearance. Loss of AQP4 polarization or mislocalization at astrocytic endfeet further compromises directional glymphatic flow, thereby promoting cerebral edema, amyloid-β retention, inflammatory amplification, and remote secondary injury. The figure illustrates the major pathological links between ischemic injury, AQP4-dependent glymphatic impairment, and downstream tissue damage.

In the progressive evolution of glymphatic dysfunction after ischemic stroke, AQP4 is a key molecule linking water transport, astrocytic responses, and reduced clearance efficiency. AQP4 is primarily localized to the perivascular endfeet of astrocytes, where it supports transmembrane water transport and the directionality of cerebrospinal fluid–interstitial fluid (CSF–ISF) exchange. Therefore, post-stroke AQP4 abnormalities should not be understood solely as changes in expression level, but rather as a dynamic process involving altered expression, loss of perivascular polarity, disruption of anchoring structures, and inflammatory responses. In the early phase of ischemia, AQP4-related changes first contribute to cerebral edema formation. Li et al. (59) showed that abnormal CSF influx and reduced AQP4 polarity occurred early after middle cerebral artery occlusion (MCAO). After reperfusion, increased AQP4 expression was accompanied by worsening edema, whereas perivascular AQP4 polarity remained impaired. This study further indicates that AQP4-related glymphatic dysfunction should be interpreted at both the expression and spatial-localization levels. Increased AQP4 expression may facilitate early water entry into astrocytes and promote edema formation, whereas loss of polarity may weaken perivascular clearance pathways, promote solute retention, and sustain glial activation and subsequent inflammatory injury. From a temporal perspective, Zhang et al. (12) further found that glymphatic transport was most markedly impaired in the early phase after stroke and partially recovered as edema resolved. Together, these studies indicate that AQP4-related glymphatic dysfunction has stage-dependent features. Increased AQP4 expression in the acute phase does not necessarily indicate enhanced clearance function; instead, it may reflect inefficient transport or abnormal influx after water-channel mislocalization. Loss of AQP4 polarity may also amplify inflammatory responses, blood–brain barrier disruption, and metabolic waste deposition. Pyroptosis can aggravate blood–brain barrier injury, AQP4 depolarization, cerebral edema, and Aβ accumulation, whereas inhibition of pyroptosis can attenuate these changes. These findings suggest that AQP4 abnormalities are not limited to fluid transport but are positioned at the intersection of neuroinflammation, barrier dysfunction, and impaired waste clearance (34, 43, 59). Accordingly, AQP4-targeted interventions should be considered in relation to disease stage. In the acute phase, the therapeutic focus may be on limiting abnormal water transport and reducing cerebral edema. During recovery, greater emphasis may be placed on restoring perivascular AQP4 localization and clearance function. Pharmacological studies further support this stage-dependent interpretation. TGN-020 may reduce cerebral edema and infarct volume in the acute phase and improve AQP4 depolarization, astrocytic responses, and neurological outcomes in the subacute phase. Related evidence also suggests that TGN-020 may act by improving glymphatic function and suppressing ERK1/2-related inflammatory and apoptotic signaling (45, 68). These findings suggest that the significance of AQP4 intervention should not be limited to anti-edema effects, but should also include the promotion of clearance, attenuation of inflammation, and improvement of cellular injury. Further evidence from transient MCAO (tMCAO) and related intervention experiments shows that post-stroke AQP4 mislocalization is accompanied by reduced CSF influx and ISF drainage. Enhancement of AQP4-M23 and SNTA1 helps restore perivascular localization, whereas increased AQP4-M1 aggravates edema and functional impairment. Taken together, these findings indicate that the core feature of AQP4-related glymphatic dysfunction is not a single change in expression level, but an imbalance in isoform composition and disruption of the anchoring complex, which together weaken its perivascular polarity. After AQP4 dissociates from astrocytic endfeet, the spatial coupling between water transport and waste clearance is disrupted. As a result, abnormal regulation of edema in the acute phase may gradually extend into insufficient clearance during the subacute and recovery phases, thereby sustaining inflammation, metabolic disturbance, and impaired neural repair (12, 67). Overall, AQP4-related glymphatic dysfunction after ischemic stroke can be summarized as a continuous process extending from an acute imbalance in water transport to subacute clearance failure. Its pathological basis includes not only altered AQP4 expression, but also loss of polarity, imbalance in isoform composition, disruption of anchoring complexes, and amplification of inflammatory signaling (**Figure 2**). Therefore, post-stroke AQP4-targeted therapy should not be reduced to continuous inhibition or simple enhancement. Instead, it should be precisely regulated according to disease stage. In the acute phase, treatment may focus on limiting abnormal water transport to control edema. In the subacute and recovery phases, therapeutic strategies should promote restoration of AQP4 polarity, recovery of perivascular anchoring, and improvement of glymphatic clearance. In this way, the therapeutic goal may extend from anti-edema treatment alone to promoting waste clearance, modulating inflammatory responses, and improving long-term neural repair (**Table 6**).

**Table 6:** Preclinical studies of ischemic stroke implicating AQP4 polarization and anchoring pathways in the regulation of glymphatic dysfunction.

Study	Model	AQP4-related focus	Principal assessment methods	Intervention	Outcome/effect
Sun et al., 2022 (34)	Rat tMCAO	AQP4 expression and polarization	MRI, apparent diffusion coefficient (ADC), AQP4-related indices	TGN-020	Edema and infarct volume↓
Yi et al., 2022 (60)	Mouse MCAO/R	AQP4 anchoring complex	CSF fluorescent tracer assay, protein analysis	Xuefu Zhuyu decoction	CSF flow improved, injury↓
Li et al., 2023 (59)	Mouse MCAO/R	AQP4–β-DG–LRP1	CSF flow assessment, Aβ quantification, transmission electron microscopy	LRIP	Recovery of lymphoid function, Aβ↓
Yang et al., 2024 (36)	Mouse MCAO/R	DP71–AQP4 axis	Efflux tracer analysis, deep cervical lymph node drainage, protein analysis	DP71 overexpression/MG132	drainage↑,Prompt DP71 as the key factor adjustment
Zhang et al., 2025 (12)	Mouse tMCAO	AQP4 M1/M23 isoforms and SNTA1	Contrast-enhanced MRI, CSF/ISF tracing, protein analysis	TGN-020/AAV-mediated modulation	Polarity lymphatic function recovery after class↑
Li et al., 2025 (66)	Mouse MCAO/R	TRPV4-dependent regulation of AQP4	Tracer studies, deep cervical lymph node drainage, MRI	HC-067047	Cerebral edema↓,Collateral vessel transport↑
Yu et al., 2025 (75)	Mouse MCAO	α1-syntrophin–AQP4 axis	Transcranial/two-photon imaging, MRI	Ketogenic diet/β-hydroxybutyrate (BHB)	Lymphoid-like function was improved

Taken together, preclinical studies of ischemic stroke suggest that post-stroke AQP4-related glymphatic dysfunction evolves from acute water-transport imbalance to loss of perivascular AQP4 polarization. This dysfunction is not governed simply by the overall abundance of AQP4, but is shaped by AQP4 mislocalization, disruption of anchoring complexes, abnormal isoform composition, and impaired regulatory signaling. Key molecules, including α-DG, β-DG, agrin, Dp71, SNTA1, α1-syntrophin, and TRPV4, influence AQP4 localization through basement membrane interactions, cytoskeletal anchoring, isoform stability, and mechanosensitive water transport. These alterations converge to disrupt CSF–ISF exchange, exacerbate brain edema, and impair metabolic waste clearance. Accordingly, therapeutic approaches should move beyond nonspecific AQP4 inhibition and instead focus on restraining maladaptive acute water flux, restoring AQP4 polarity, and preserving perivascular anchoring architecture to facilitate glymphatic recovery after stroke.

### 5.3 Meningeal lymphatics and border-associated immune responses after ischemic stroke

**Figure 3:**
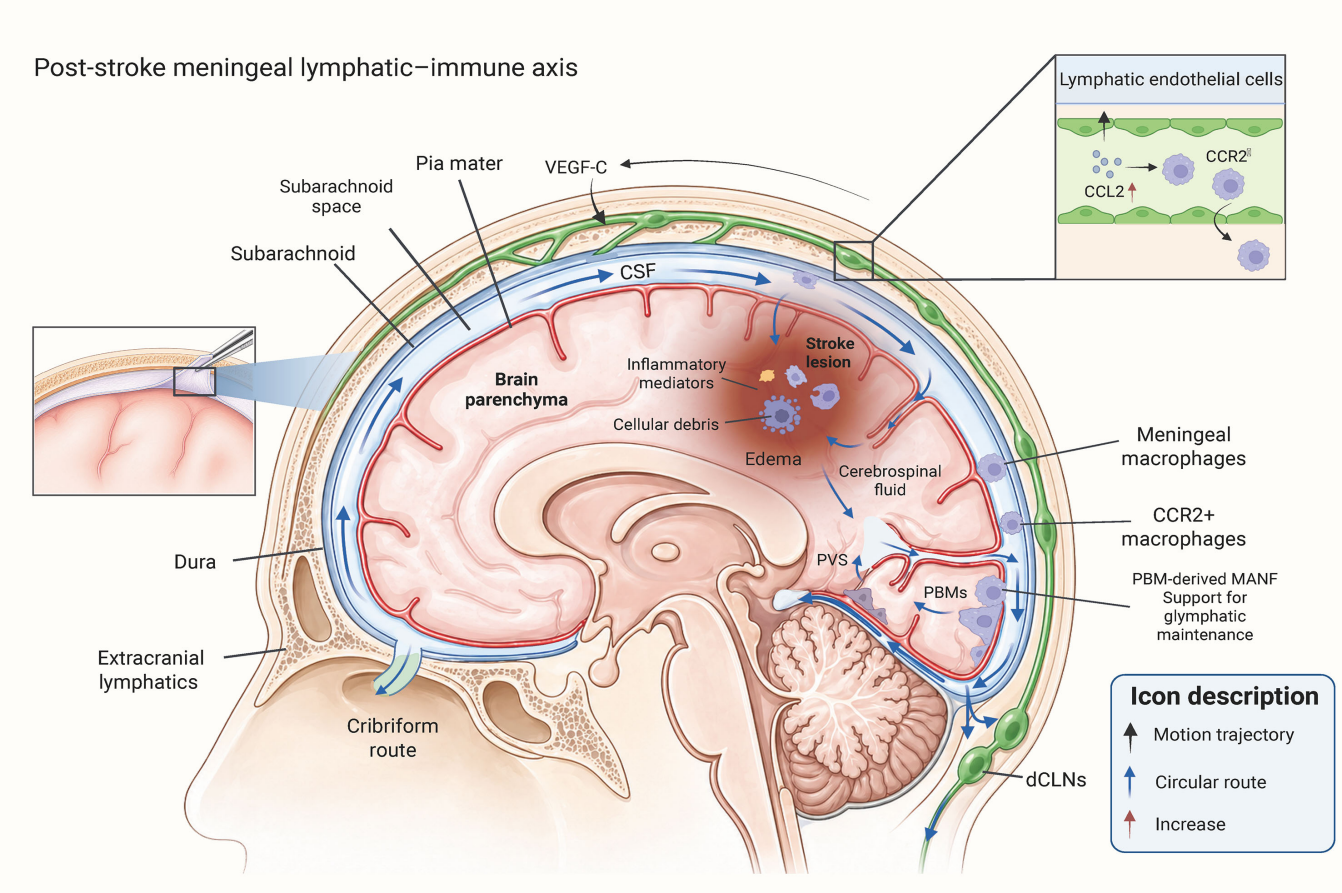
Meningeal immune–lymphatic interface after ischemic stroke. Ischemic stroke activates a meningeal immune–lymphatic interface in which border-associated macrophages, recruited monocytes, and other immune cells interact with meningeal lymphatic drainage pathways. Meningeal macrophage activation, migration, and potential perivascular entry may contribute to neuroinflammatory signaling at the brain–border interface. Meanwhile, meningeal lymphatic vessels drain cerebrospinal fluid, inflammatory mediators, antigens, and immune cells to the deep cervical lymph nodes (dCLNs), thereby connecting intracranial injury with peripheral immune responses. VEGF-C-driven lymphatic remodeling and CCL2–CCR2-mediated monocyte/macrophage recruitment represent potential regulatory nodes within this system. This schematic illustrates how meningeal immunity and lymphatic drainage may jointly shape post-ischemic neuroinflammation and secondary injury progression.

AQP4 remodeling mainly explains impaired cerebrospinal fluid–interstitial fluid (CSF–ISF) exchange within the brain parenchyma. Whether this fluid and inflammatory burden can be further cleared from the brain depends on meningeal lymphatic vessels and their downstream drainage pathway to the deep cervical lymph nodes. Therefore, post-stroke meningeal lymphatics should not be regarded as an independent structure separate from the glymphatic system. Rather, they represent the extracranial outlet of the intracerebral clearance pathway, whereas immune cells at the meningeal borders serve as cellular effectors of this outlet by participating in metabolic waste handling, inflammatory-cell trafficking, and resolution of immune responses. Within this efflux and immune-communication pathway, Boisserand et al. (69) found that meningeal lymphatic vessels may undergo transient dilation in the early phase after stroke. VEGF-C pretreatment more stably preserved lymphatic structures, enhanced CSF drainage to the deep cervical lymph nodes, and improved functional outcomes. However, this protective effect was no longer evident when afferent drainage to the deep cervical lymph nodes was blocked or when VEGF-C was administered acutely after stroke. These findings suggest that endogenous remodeling of meningeal lymphatics after ischemia may represent a transient compensatory response that is insufficient to sustain the long-term clearance of fluid, cellular debris, and inflammatory signals. Its influence on stroke outcomes may depend on whether the meningeal lymphatic–deep cervical lymph node axis is mature and patent enough to convert intracerebral clearance burden into peripheral lymph node–mediated immune regulation. Thus, meningeal lymphatic dysfunction reflects not only insufficient drainage but also delayed termination of border-associated immune responses, potentially contributing to persistent inflammation and limited recovery. Keuters et al. (51) further supported the potential value of enhancing meningeal lymphatic drainage. After VEGF-C-induced growth of meningeal lymphatic vessels, CSF–ISF drainage was improved, inflammation was reduced, and neurological function was enhanced. However, the effects on acute infarct volume and cerebral edema were relatively limited. These findings suggest that the major effect of meningeal lymphatic intervention may not be the direct reversal of acute ischemic injury. Instead, it may influence stroke outcomes by improving fluid dynamics, promoting inflammation resolution, and supporting repair during the recovery phase. Meningeal lymphatic function is also closely linked to border-associated immune-cell trafficking. Meningeal lymphatics can recruit and transport macrophages to cervical lymph nodes through the CCL2–CCR2 axis. Blocking this pathway aggravates inflammation, edema, and functional impairment, whereas enhancing drainage ameliorates these injuries (10). These findings indicate that meningeal lymphatics are not passive drainage conduits, but active regulatory platforms involved in immune-cell migration and inflammation resolution. In other words, their protective effects may arise from the combined actions of fluid clearance and immune-cell efflux. Border-associated immune cells may also participate in chronic post-stroke clearance and cognitive protection. Li et al. (13) showed that border-associated macrophages undergo sustained changes after stroke and help maintain glymphatic flow and extracerebral drainage. Insufficient border-associated macrophage function in the chronic phase can aggravate Aβ deposition and cognitive impairment, whereas MANF supplementation can promote Aβ clearance and improve cognitive outcomes. These findings suggest that border-associated immune cells participate not only in acute inflammatory regulation but also in long-term metabolic waste clearance and cognitive recovery after stroke. Taken together, meningeal lymphatics and border-associated immunity together constitute the efflux and immune-regulatory unit of the glymphatic clearance pathway after ischemic stroke. Their roles include not only CSF–ISF and metabolic waste efflux, but also inflammatory-cell trafficking, immune-response resolution, and long-term cognitive protection. Therefore, future interventions should not treat meningeal lymphatics simply as passive drainage conduits. Instead, studies should simultaneously assess meningeal lymphatic structural maturation, the status of border-associated immune cells, and therapeutic timing. More precise targeting of the meningeal lymphatic–deep cervical lymph node efflux axis and its immune-cell regulatory functions may provide a new therapeutic direction for reducing persistent post-stroke inflammation, promoting waste clearance, and improving long-term functional recovery (**Figure 3**).

### 5.4 Interventional studies targeting glymphatic and meningeal immune pathways

If post-stroke clearance impairment is understood as a continuous imbalance among intraparenchymal fluid exchange, meningeal lymphatic efflux, and border-associated immune regulation, interventional studies should not be limited to a single molecular target. Instead, they should focus on phase-specific remodeling of this clearance and immune-regulatory network. Existing evidence is derived mainly from animal models and involves multiple strategies, including pharmacological modulation, metabolic interventions, physical stimulation, and enhancement of meningeal lymphatics. Although these interventions differ in approach, they converge on a central concept: post-stroke glymphatic and meningeal immune pathways are not static damaged structures, but dynamic functional units that continue to change during edema formation, inflammatory amplification, waste retention, and repair. In the acute phase, TGN-020 administration or VEGF-C-related pretreatment can influence cerebral edema, infarct injury, and neurological function (34, 69). Studies of AQP4 isoforms and SNTA1 also indicate that improvement in glymphatic function is reflected more by restoration of perivascular clearance structures and organized flow direction than by changes in channel expression alone (12). These findings have shifted the focus of interventional studies from inhibition of a single pathological process toward integrated regulation aimed at maintaining the directionality of fluid exchange, promoting efflux of inflammatory mediators, and improving the continuity of intracerebral and extracerebral clearance. Metabolic and combined interventions have further expanded this concept. β-hydroxybutyrate and ketogenic diet can improve glymphatic flow and attenuate edema-related injury after stroke, whereas delayed β-hydroxybutyrate administration can still improve clearance function without markedly reducing infarct volume (75). These findings suggest that enhancement of clearance and salvage of the ischemic core may not share the same therapeutic time window. Clearance-promoting strategies may be more relevant to microenvironmental remodeling during the subacute and recovery phases, including edema resolution, removal of inflammatory products, and efflux of metabolic waste. Other interventions, including remote ischemic postconditioning, Xuefu Zhuyu decoction, and Yizhi Fangdai formula, have also been reported to improve CSF–ISF exchange, reduce Aβ-related burden, or alleviate inflammatory cell death (59, 60, 70). Taken together, these studies suggest that targeting glymphatic pathways is not limited to altering fluid velocity. Rather, such interventions may reshape the post-stroke local immune microenvironment by influencing blood–brain barrier stability, glial responses, perivascular structures, and clearance of inflammatory mediators. Compared with single-target modulation, these multilayered effects may better reflect the complex pathology of post-stroke secondary injury. Physical stimulation studies provide another approach for regulating clearance pathways. Combined electro-optical stimulation improved functional outcomes and reduced inflammatory mediator accumulation in an MCAO/R model, accompanied by recovery of glymphatic function. Low-intensity focused ultrasound has shown the ability to modulate local CSF influx, and repeated stimulation improved bilateral impairment of CSF influx after stroke (50, 61). Compared with pharmacological interventions, these approaches have adjustable stimulation sites, frequencies, and durations, making them potentially more compatible with neuromodulatory strategies during stroke rehabilitation. However, glymphatic flow is strongly phase dependent. Excessive CSF influx in the hyperacute phase may contribute to tissue swelling, whereas restoration of directed flow during the subacute and recovery phases may facilitate waste clearance and inflammation resolution (69). Therefore, when evaluating the effects of physical stimulation, it is insufficient to focus only on enhanced CSF influx. Efflux efficiency, drainage to the deep cervical lymph nodes, meningeal immune-cell migration, and long-term cognitive and motor outcomes should also be assessed. Only by examining influx, exchange, and efflux as a continuous process can the significance of these interventions for stroke recovery be more accurately interpreted. Studies targeting meningeal lymphatic and meningeal immune pathways more directly highlight the peripheral connectivity of the clearance system. VEGF-C can promote the growth of meningeal lymphatic vessels and enhance drainage to the deep cervical lymph nodes, accompanied by reduced neuroinflammation and improved functional outcomes. Keuters et al. (51) found that the protective effect disappeared when afferent drainage to the deep cervical lymph nodes was blocked, indicating that the integrity of the meningeal lymphatic efflux axis is an important basis for these benefits. Studies of dural lymphangiogenesis have also shown that AAV9-mediated VEGF-C can enhance CSF and ISF drainage and improve selected functional outcomes, although its effects on acute infarct volume and cerebral edema are relatively limited. These findings suggest that enhancement of meningeal lymphatics may be better understood as a regulatory strategy that promotes inflammatory resolution and reconstruction of the repair microenvironment, rather than as a means of directly reversing early ischemic core injury. The CCL2–CCR2 axis between meningeal lymphatics and macrophages further indicates that the meningeal lymphatic system is not merely a drainage channel, but a dynamic interface involved in immune-cell recruitment, trafficking, and clearance. Therefore, evaluation of interventions targeting meningeal immune pathways should not be limited to morphological changes in lymphatic vessels. It should also incorporate immune-cell migration, inflammatory phenotype, deep cervical lymph node responses, and systemic immune status. Current evidence suggests that the main significance of targeting glymphatic and meningeal immune pathways lies in regulating phase-specific clearance impairment after stroke. Acute-phase interventions may focus more on limiting abnormal fluid flux and edema formation, whereas subacute- and recovery-phase interventions may place greater emphasis on metabolic waste efflux, inflammation resolution, and restoration of immune homeostasis. At the same time, studies indicate that effective clearance through intraparenchymal CSF–ISF exchange depends on the integrity of downstream meningeal lymphatic efflux and deep cervical lymph node pathways (10). The role of meningeal immune cells is also influenced by cell type, disease stage, and the local microenvironment, and therefore cannot be simply classified as proinflammatory or protective. Future studies should interpret interventional effects in relation to stroke subtype, disease stage, drainage direction, and immune status, and should avoid equating enhanced clearance directly with neuroprotection. At present, strategies targeting glymphatic and meningeal immune pathways remain largely at the stage of mechanistic investigation and early translational exploration. Clinical translation should proceed cautiously and only after efficacy, safety, and appropriate target populations have been adequately validated. Differences between rodents and humans in meningeal lymphatic anatomy, CSF dynamics, brain volume, immune composition, and vascular risk burden may influence the extrapolation of animal findings to patients with clinical stroke. Different stroke subtypes also differ in their dominant pathological burden, clearance requirements, and therapeutic time windows. Therefore, the same intervention may have distinct biological effects and clinical implications in ischemic stroke, intracerebral hemorrhage, and subarachnoid hemorrhage. Ischemic stroke involves post-infarct edema, abnormal AQP4 polarization, and clearance of inflammatory mediators; intracerebral hemorrhage emphasizes clearance of hematoma products, iron deposition, and perihematomal edema; and subarachnoid hemorrhage mainly affects the CSF compartment, meningeal spaces, and meningeal lymphatic endothelium. Thus, strategies that promote glymphatic flow, meningeal lymphangiogenesis, or immune-cell trafficking may exert subtype- and stage-specific effects. Future studies should integrate standardized models, multimodal imaging, biomarkers, long-term functional outcomes, and systematic safety assessment to define their translational value and applicable boundaries.

## 6 Preclinical evidence for altered glymphatic pathways and meningeal lymphatic drainage after hemorrhagic stroke

### 6.1 Glymphatic dysfunction and altered meningeal lymphatic drainage in intracerebral hemorrhage

Building on evidence from ischemic stroke regarding glymphatic clearance impairment and meningeal immune responses, hemorrhagic stroke further highlights the interconnections among intracranial fluid exchange, elimination of blood-derived components, and immune regulation. Preclinical studies suggest that clearance of hematoma products and inflammatory mediators depends not only on local phagocytes, but also on CSF–ISF exchange, AQP4-mediated transport, and meningeal lymphatic drainage to the deep cervical lymph nodes (46, 76). Within this clearance pathway, Tsai et al. (47) showed that meningeal lymphangiogenesis and drainage to the deep cervical lymph nodes are mainly enhanced during the recovery phase after intracerebral hemorrhage. Ablation of meningeal lymphatic vessels or blockade of deep cervical lymph node drainage delayed hematoma absorption, whereas enhancement of meningeal lymphatic function reduced residual hematoma, iron deposition, and glial responses, and improved neurological function. This finding indicates that glymphatic–meningeal lymphatic dysfunction after intracerebral hemorrhage is not merely a disorder of fluid drainage, but also reflects restricted elimination of hematoma degradation products, iron burden, and inflammatory mediators. Meningeal lymphatics, therefore, serve as a key outlet linking hematoma clearance, control of iron toxicity, and resolution of secondary inflammation. Functional insufficiency of this pathway may contribute to delayed hematoma absorption and persistence of long-term neurological injury. On this basis, Yu et al. (72) further showed that Panax notoginseng saponins improved hematoma absorption and functional recovery by promoting meningeal lymphatic drainage and lymphangiogenesis. These effects were attenuated after lymphatic vessel ablation, suggesting that an intact meningeal lymphatic network may be essential for interventions designed to promote hematoma clearance. From the perspective of non-pharmacological intervention, repetitive transcranial magnetic stimulation improved glymphatic and meningeal lymphatic function after intracerebral hemorrhage, enhanced clearance of tracers from the brain parenchyma, and promoted behavioral recovery. These findings suggest a potentially modifiable functional coupling among neural activity, cerebral hemodynamics, and intracranial drainage (62). From a circadian perspective, melatonin restored glymphatic transport after intracerebral hemorrhage, promoted absorption of hematoma and cerebral edema, and attenuated blood–brain barrier injury. Its protective effects were partially reduced by a melatonin receptor antagonist, with AQP4 polarization and rhythmic fluid exchange also involved (77). The study by Li et al. (35) further supports this view, showing that enhancement of AQP4-related function can improve edema, blood–brain barrier disruption, inflammatory responses, and glymphatic clearance. These findings indicate that AQP4 is not an isolated water-channel molecule, but is more likely positioned at the intersection of astrocytic state, blood–brain barrier integrity, inflammatory amplification, and intracerebral waste clearance (49). In aggregate, clearance impairment after intracerebral hemorrhage involves two continuous components: AQP4-dependent CSF–ISF exchange and meningeal lymphatic drainage to the deep cervical lymph nodes. The former influences the efficiency with which perihematomal fluid and metabolic products enter efflux pathways, whereas the latter determines whether these pathological products can leave the cranial cavity. Therefore, glymphatic dysfunction and meningeal lymphatic remodeling after intracerebral hemorrhage may represent alterations at different levels of the same clearance network. Enhancing this continuous drainage pathway may promote hematoma absorption, reduce edema and neuroinflammation, and improve functional recovery. Future studies should clarify the temporal relationship between these components and validate the therapeutic value and translational feasibility of targeting AQP4 or the meningeal lymphatic system at different disease stages.

### 6.2 Alterations in meningeal lymphatic function after subarachnoid hemorrhage and in related models

**Figure 4:**
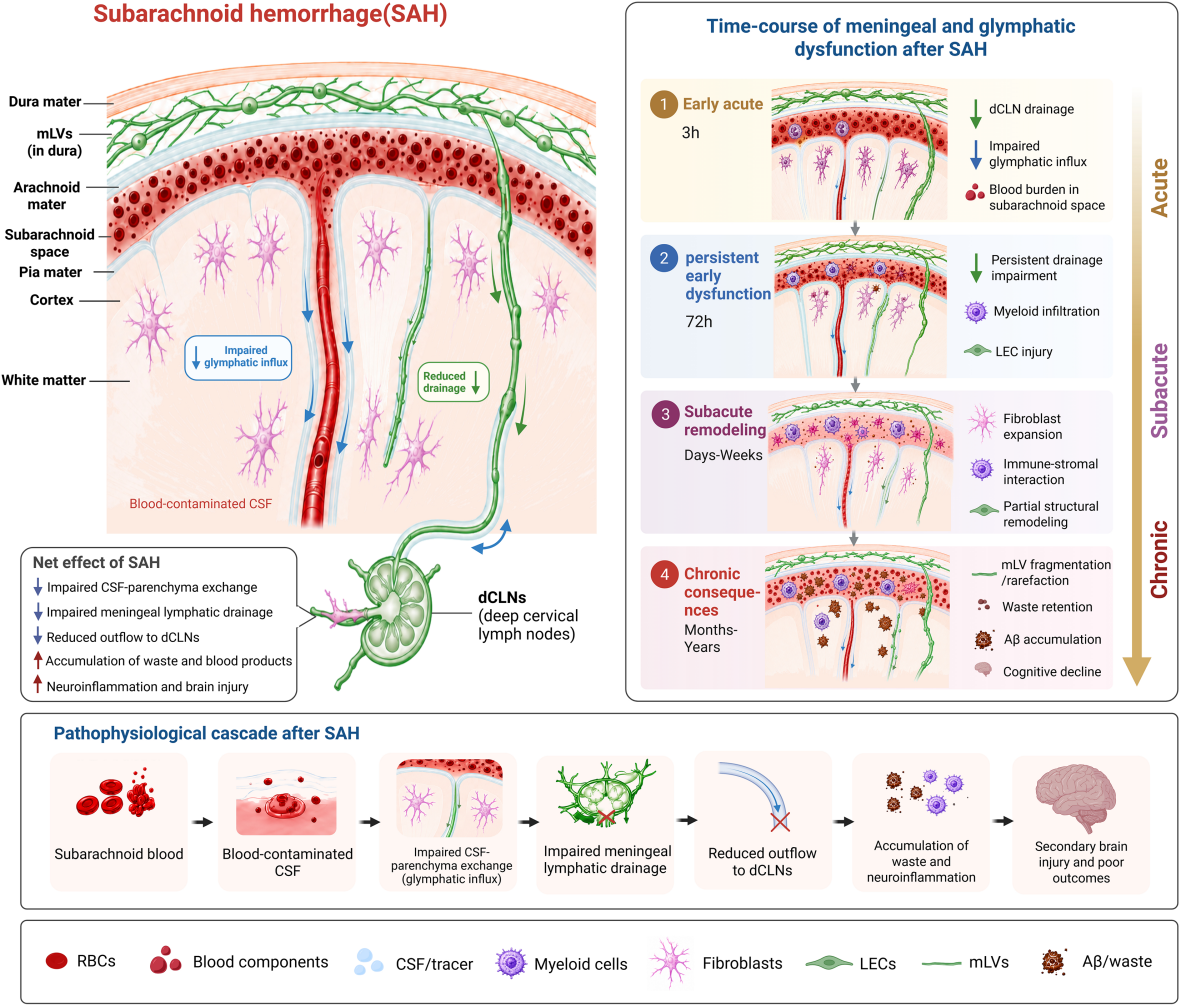
Proposed mechanism linking meningeal lymphatic dysfunction and recovery to hemorrhagic stroke outcomes. The figure illustrates evidence-supported mechanisms by which meningeal lymphatic vessels (mLVs) may participate in hemorrhagic stroke pathology and recovery. In intracerebral hemorrhage (ICH), blood-product accumulation after hematoma formation promotes edema and inflammation. VEGF-C–associated enhancement or restoration of mLV function may improve the clearance of blood-derived components and support functional recovery. In subarachnoid hemorrhage (SAH), erythrocytes and blood metabolites accumulate within the subarachnoid space, where they may directly damage the meningeal lymphatic network. mLV injury or fragmentation can impair erythrocyte and metabolite drainage, thereby exacerbating inflammation and contributing to delayed cognitive dysfunction. By emphasizing mLV injury/repair, blood and erythrocyte clearance, edema, inflammation, and cognition, this schematic highlights why hemorrhagic stroke should be interpreted through a meningeal lymphatic pathway framework rather than being subsumed entirely under ischemic stroke–derived glymphatic models.

Unlike intraparenchymal hemorrhage, subarachnoid hemorrhage (SAH) directly affects the cerebrospinal fluid (CSF) and meningeal compartments. It is therefore particularly likely to disrupt CSF circulation, perivascular glymphatic transport, and meningeal lymphatic drainage to the deep cervical lymph nodes. Gaberel et al. (52) demonstrated that SAH impairs glymphatic influx and intracerebral clearance, whereas tissue-type plasminogen activator partially restores these processes. These findings suggest that blood components or fibrin deposition may obstruct perivascular pathways. Evidence from non-human primates further supports this concept (65), indicating that SAH-induced impairment of CSF–parenchymal exchange is not restricted to rodent models but also occurs in gyrencephalic brains that more closely resemble the human brain. Thus, blood-mediated obstruction of perivascular routes may represent an important translational mechanism contributing to clearance failure and delayed brain injury after SAH. As research has shifted from fluid perfusion to lymphatic structures themselves, injury to the meningeal lymphatic endothelium has received increasing attention. Wang et al. (63) reported that meningeal lymphatic architecture is disrupted early after SAH and that drainage to the deep cervical lymph nodes is reduced. This process was mechanistically associated with THBS1–CD47-mediated apoptosis of meningeal lymphatic endothelial cells. Together, these observations indicate that impaired clearance after SAH is not simply a consequence of reduced CSF flow. Rather, it reflects a continuum of efflux failure involving both obstruction of perivascular pathways and damage to meningeal lymphatic outflow routes. After blood enters the subarachnoid space, it may interfere directly with fluid exchange within the brain parenchyma while also injuring the meningeal lymphatic endothelium. These changes may limit the timely transport of blood-breakdown products, inflammatory mediators, and immune cells to cervical lymph nodes. Accordingly, SAH-associated meningeal lymphatic dysfunction may represent an important mechanistic link between early CSF circulatory disturbance, persistent inflammation, and delayed brain injury. Additional studies have shown that blockade of deep cervical lymphatic drainage aggravates brain injury, whereas VEGF-C promotes erythrocyte clearance and improves neurological outcomes (71). These findings support the view that the meningeal lymphatic system is not only an efflux route for CSF, but may also serve as a regulatory pathway for the clearance of blood-derived components and the control of secondary injury. Meningeal lymphatic impairment is closely associated with remodeling of the meningeal immune microenvironment. During the acute phase, myeloid-cell infiltration and inflammatory responses may compromise lymphatic function. During the subacute phase, fibroblast expansion may contribute to tissue repair. Placental growth factor, an important ligand in the VEGF signaling network, may participate in early lymphatic repair after inflammation (64). This dynamic process suggests that meningeal lymphatic dysfunction is not a single structural lesion, but rather a stage-dependent pathological process involving immune-cell recruitment, stromal-cell responses, and lymphatic endothelial repair. Meningeal lymphatic dysfunction may also contribute to long-term cognitive impairment after SAH. Cai et al. (78) found that SAH induces fragmentation and atrophy of meningeal lymphatic vessels, leading to impaired drainage of CSF and interstitial fluid, metabolite accumulation, and delayed cognitive decline. VEGF-C preserved meningeal lymphatic vessels, reduced amyloid-β deposition in the hippocampal CA1 region, and improved cognitive performance, potentially through activation of PI3K–AKT signaling. Related intraventricular hemorrhage models further support this mechanism. In adult and neonatal rodents, erythrocytes can be cleared through meningeal lymphatic vessels to the deep cervical lymph nodes. Erythrocytes have also been observed within meningeal lymphatic vessels in postmortem meningeal tissue from patients with fatal intraventricular hemorrhage. Transcranial near-infrared light stimulation enhanced meningeal lymphatic clearance, promoted the efflux of erythrocytes and macromolecules from the ventricular system, and improved recovery (79). In summary, studies of SAH and related hemorrhage models indicate that, once blood enters the CSF compartment, it can directly impair CSF circulation and perivascular glymphatic transport while also inducing meningeal immune-cell infiltration, lymphatic endothelial injury, and reduced lymphatic drainage. When meningeal lymphatic function is compromised, the clearance of erythrocytes, hemoglobin-derived products, inflammatory mediators, and metabolic waste is impeded. This may further exacerbate cerebral edema, neuroinflammation, delayed brain injury, and cognitive dysfunction (**Figure 4**). Future studies should further distinguish the mechanisms involved in acute blood clearance, subacute immune regulation, and chronic cognitive protection, and should evaluate the safety and efficacy of strategies that enhance meningeal lymphatic function across distinct therapeutic windows (**Table 7**).

**Table 7:** Preclinical evidence on meningeal immunity and meningeal lymphatic drainage in ischemic and hemorrhagic stroke.

Study	Stroke type and model	Primary focus	Main intervention or comparator	Major findings
Yang et al., 2024 (44)	Ischemic stroke; rat MCAO model	mLVs, dCLNs, and peripheral immunity	dCLN excision; AAV-VEGF-C treatment	Meningeal lymphatic drainage modulates post-stroke immune responses
Yu et al., 2026 (38)	Ischemic stroke; single-cell profiling with experimental validation	BAMs, TNF, STAT3	BAM characterization; BAM depletion	BAMs represent an important source of neuroinflammatory signaling after stroke
Pedragosa et al., 2018 (39)	Ischemic stroke; rat tMCAO model and human brain tissue	CD163+ BAMs	Cell sorting; macrophage depletion	BAMs amplify acute inflammation and vascular injury after ischemic stroke
Bai et al., 2022 (40)	Ischemic stroke; rat MCAO model	Meningeal lymphatic drainage and T cells	Calvarial distraction; lymphatic ablation	Meningeal lymphatic function can be mechanically modulated after stroke.
Rajan et al., 2020 (42)	Ischemic stroke; rodent models and human brain tissue	BAM phenotype and origin	RNA sequencing; chimeric mouse models	BAMs are dynamic border-associated immune cells that undergo phenotypic remodeling after stroke
Keuters et al., 2025 (51)	Ischemic stroke; mouse tMCAO model	dLVs, VEGF-C, and CSF/ISF drainage	AAV-VEGF-C	VEGF-C–mediated lymphatic enhancement may promote post-stroke recovery
Yanev et al., 2020 (56)	Ischemic stroke; photothrombotic and tMCAO mouse models	Meningeal lymphatic development and VEGFR3 signaling	Cross-model comparison; Vegfr3 mutation	The response of meningeal lymphatics varies according to the experimental stroke model
Riew et al., 2022 (57)	Ischemic stroke; rat MCAO model	Meningeal macrophages and Virchow–Robin spaces	MCAO versus sham surgery	The study suggests a potential route by which meningeal immune cells enter the brain after stroke
Wang et al., 2026 (10)	Ischemic stroke; mouse MCAO model	mLVs, macrophages, and the CCL2–CCR2 axis	CCR2 inhibition; VEGF-C treatment	The findings link immune-cell clearance to meningeal lymphatic drainage after stroke
Li et al., 2025 (13)	Ischemic stroke; distal MCAO mouse model	PBMs, Aβ clearance, and MANF	PBM depletion; MANF supplementation	PBMs are associated with waste clearance and cognitive outcomes after stroke
Boisserand et al. (69),	Ischemic stroke; mouse tMCAO model	VEGF-C, MLVs, and dCLN drainage	AAV-VEGF-C pretreatment; lymphatic blockade	Therapeutic efficacy depends on the treatment window and an intact lymphatic drainage pathway
Tsai et al., 2022 (47)	Intracerebral hemorrhage; mouse model	mLVs, dCLNs, and hematoma clearance	mLV ablation; dCLN blockade; VEGF-C or pharmacological enhancement	mLVs are potential therapeutic targets for hematoma resolution after ICH
Luo et al., 2024 (71)	Subarachnoid hemorrhage; rat and rabbit models	Cervical lymphatic vessels and erythrocyte clearance	dCLV ligation; VEGF-C treatment	Meningeal lymphatics contribute to blood clearance after SAH
Wang et al., 2023 (63)	Subarachnoid hemorrhage; mouse model	mLV injury and mLEC apoptosis	Single-cell and spatial transcriptomics; THBS1–CD47 validation	The study identifies mechanisms underlying mLV injury after SAH
Zhu et al., 2025 (64)	Subarachnoid hemorrhage; mouse model	Meningeal immune microenvironment, fibroblasts, and mLECs	Single-cell and spatial transcriptomic analyses	mLV function dynamically changes with the meningeal microenvironment after SAH
Cai et al., 2026 (78)	Subarachnoid hemorrhage; mouse model	mLV integrity, CSF/ISF drainage, and cognition	mLV ablation; VEGF-C treatment	The findings link mLV dysfunction to long-term cognitive sequelae after SAH

Preclinical evidence indicates that the meningeal immune system and meningeal lymphatic drainage are important modulators of post-stroke pathology in both ischemic and hemorrhagic settings. In ischemic stroke, meningeal lymphatic vessels and deep cervical lymph nodes mediate the peripheral drainage of brain-derived antigens, inflammatory mediators, and cerebrospinal/interstitial fluid, thereby influencing neuroinflammation, systemic immune responses, and neurological recovery. Meningeal border-associated macrophages also participate in vascular repair and immune regulation through phagocytosis, cytokine production, and phenotypic remodeling. In hemorrhagic stroke and subarachnoid hemorrhage, the meningeal lymphatic system contributes to the clearance of erythrocytes, hematoma-related products, and inflammatory debris, whereas its dysfunction may worsen brain edema, neuroinflammation, and cognitive impairment. Moreover, VEGF-C/VEGFR3-mediated enhancement of meningeal lymphangiogenesis or lymphatic drainage may facilitate the removal of toxic metabolites and inflammatory mediators, mitigate neurological injury, and improve outcomes. These findings support the meningeal immune–lymphatic axis as a central mechanism of post-stroke inflammation, waste clearance, and functional recovery, and as a promising therapeutic target in stroke.

## 7 Discussion

Stroke is associated with high incidence, substantial disability, and a prolonged disease burden. Its clinical consequences extend beyond acute neural tissue injury and include motor impairment, cognitive decline, affective disturbances, fatigue, and impaired long-term functional recovery. Understanding the interplay among impaired cerebral waste clearance, disrupted fluid homeostasis, and sustained inflammatory responses after stroke is therefore essential for explaining long-term outcomes. Glymphatic-related imaging biomarkers provide a visual window into these processes. Integrating these biomarkers with meningeal lymphatic drainage, migration of border-associated immune cells, and peripheral immune status may help delineate, at a systems level, the continuum of impaired clearance, persistent inflammation, and limited recovery after stroke.

Within this systems-level framework, it is essential to distinguish the aspects of the current evidence base supported by clinical imaging studies from those supported primarily by preclinical mechanistic data. Clinical studies using DTI-ALPS, enlarged perivascular spaces, choroid plexus volume, free-water imaging, and MRI-based indices of neurovascular coupling have shown that glymphatic-related abnormalities are associated with stroke severity, cognitive and motor function, neuropsychiatric symptoms, and long-term prognosis. These findings provide patient-level imaging phenotypes of impaired post-stroke clearance and suggest potential clinical relevance. However, these measures remain indirect imaging biomarkers and cannot directly capture AQP4 polarization, meningeal lymphatic drainage, immune-cell migration, or molecular waste clearance. In contrast, preclinical studies using tracer imaging, histology, molecular assays, and interventional experiments can more directly delineate impaired CSF–interstitial fluid exchange, altered AQP4 localization, disrupted meningeal lymphatic drainage to deep cervical lymph nodes, and the role of border-associated immune-cell responses in cerebral edema formation, persistent neuroinflammation, and tissue repair. Thus, current clinical evidence mainly supports associations between imaging abnormalities and stroke outcomes, whereas the underlying cellular and molecular basis remains largely interpreted through experimental models.

Given this distinction in evidence, post-stroke alterations in glymphatic function and meningeal immunity are best understood as an integrative framework supported jointly by clinical phenotypes and experimental mechanisms. Clinical studies provide imaging phenotypes, disease associations, and prognostic signals in real-world patient populations. Preclinical studies, in turn, use controlled models and mechanistic interventions to explain the cellular and molecular basis underlying imaging abnormalities and generate testable hypotheses. These two lines of evidence are complementary. Together, they help avoid overinterpreting indirect imaging markers as definitive mechanisms while also preventing findings from animal experiments from being detached from the clinical context of human stroke. Among these mechanisms, AQP4 represents a key node linking imaging phenotypes with mechanistic interpretation. Although current clinical imaging cannot directly measure AQP4 polarization or astrocytic endfoot architecture, preclinical evidence indicates that abnormal AQP4 expression, loss of perivascular localization, and impaired polarization may influence the directionality of CSF–interstitial fluid exchange, cerebral edema formation, metabolic waste clearance, and resolution of inflammation. Future studies should strengthen bidirectional translation by aligning tracer-based measurements, AQP4 localization, and molecular and immunological readouts from animal models with MRI-based indices in human cohorts. Such studies should also account for stroke subtype, lesion location, reperfusion status, and long-term clinical outcomes to clarify which findings are already clinically supported and which mechanisms remain primarily based on experimental models.

In summary, post-stroke clearance impairment should not be viewed solely as defective fluid exchange within the brain parenchyma. Instead, it should be understood as a coordinated disturbance involving glymphatic transport, AQP4-mediated regulation of perivascular architecture, meningeal lymphatic outflow, and border-associated immune responses. This framework provides a more coherent explanation for edema formation, solute retention, persistent inflammation, and limited functional recovery after ischemic stroke. It may also help distinguish subtype-specific mechanisms in hemorrhagic stroke, including impaired clearance of blood-derived products, obstruction of cerebrospinal fluid circulation, and meningeal lymphatic injury. At present, however, the available evidence is insufficient to define this pathway as an independent determinant of stroke prognosis or as a universal therapeutic target. The glymphatic–meningeal immune axis is therefore best regarded as an integrative analytical framework for interpreting post-stroke clearance failure and sustained secondary injury, rather than as a single decisive mechanism. Future studies should stratify analyses by stroke subtype, disease stage, and multimodal biomarkers to clarify their dynamic evolution, clinical relevance, and therapeutic potential.

## 8 Strengths and limitations

A major strength of this review is its mechanism-integrated perspective, which considers glymphatic transport, meningeal lymphatic drainage, and meningeal immune responses within a shared framework of post-stroke secondary brain injury and functional recovery. By integrating these lines of evidence, this review emphasizes that cerebral edema, metabolic waste retention, persistent neuroinflammation, and limited recovery after stroke may not be isolated pathological events. Instead, they may reflect a broader disturbance involving the brain parenchyma, perivascular spaces, meningeal borders, and peripheral lymphatic drainage. Clinical studies using DTI-ALPS, enlarged perivascular spaces, choroid plexus volume, free-water imaging, and MRI-based indices of neurovascular coupling suggest that glymphatic-related imaging abnormalities are associated with stroke severity, motor function, cognitive impairment, neuropsychiatric symptoms, fatigue, and prognosis. These findings provide patient-level imaging evidence of impaired post-stroke clearance. In parallel, preclinical studies using tracer imaging, histology, molecular assays, and interventional experiments have identified mechanistic substrates involving impaired CSF–interstitial fluid exchange, abnormal AQP4 polarization, disrupted meningeal lymphatic drainage, and border-associated immune-cell responses. Considering these two categories of evidence together helps clarify which imaging phenotypes and clinical associations are supported by clinical data and which cellular and molecular mechanisms remain primarily inferred from experimental models. This approach strengthens the hierarchical interpretation of the evidence and supports the translational logic of the review. Another important strength of this review is its organization by stroke subtype, disease stage, and pathway category. Ischemic stroke, intracerebral hemorrhage, and subarachnoid hemorrhage may all involve impaired clearance and activation of meningeal immunity, but their dominant pathological burdens differ. Ischemic stroke is more prominently associated with abnormal CSF influx, restricted interstitial fluid efflux, AQP4 mislocalization, cerebral edema, and amplified inflammation. Hemorrhagic stroke, in contrast, is more closely related to hematoma-derived products, erythrocytes, hemoglobin-related substrates, iron deposition, pathway obstruction, meningeal lymphatic injury, and delayed cognitive impairment. This stratified analysis avoids reducing glymphatic and meningeal immune alterations across distinct stroke subtypes to a single uniform process. It also aligns mechanistic interpretation more closely with subtype-specific pathological features and provides clearer directions for future subtype-specific studies. Given the substantial heterogeneity among the included studies in imaging metrics, animal models, observational windows, and outcome measures, narrative synthesis rather than quantitative pooling is more appropriate for the current evidence base. This approach also preserves the complexity and interdisciplinary nature of mechanistic research in this field.

Several limitations should be considered when interpreting the available evidence. Most clinical studies rely on indirect imaging biomarkers, including DTI-ALPS, enlarged perivascular spaces, and related MRI indices. Although these measures are clinically accessible and may reflect patient-level phenotypes of altered fluid dynamics and structural fluid retention after stroke, they cannot directly measure AQP4 polarization, meningeal lymphatic drainage, immune-cell migration, or molecular waste clearance. Therefore, current clinical evidence mainly supports associations between glymphatic-related imaging abnormalities and stroke outcomes. The potential causal mechanisms underlying these associations require further validation using multimodal imaging, fluid biomarkers, and longitudinal clinical studies. Evidence for interventions targeting glymphatic and meningeal immune pathways remains largely derived from animal models and early experimental studies. Differences between rodents and humans in meningeal lymphatic anatomy, CSF dynamics, brain volume, immune composition, and vascular risk factor burden may limit the direct extrapolation of these findings to clinical stroke populations. The effects of strategies targeting AQP4 regulation, VEGF-C-mediated meningeal lymphangiogenesis, physical stimulation, and immune-cell trafficking may also vary according to stroke subtype, lesion location, reperfusion status, and disease stage. Future studies should systematically evaluate potential safety concerns, including altered blood–brain barrier permeability, inflammatory imbalance, aberrant immune activation, and long-term structural remodeling. Standardized models, subtype-stratified analyses, and well-designed clinical studies are needed to further define the translational value and applicable boundaries of these strategies.

As this field continues to evolve, future research should establish standardized multimodal imaging protocols and prospective longitudinal clinical cohorts. Tracer-based, molecular, and immunological readouts from animal experiments should be aligned with MRI-based indices in human populations. These analyses should be further stratified by blood and CSF biomarkers, stroke subtype, disease stage, and reperfusion status. Through such bidirectional integration of clinical and preclinical evidence, the true value of glymphatic and meningeal immune pathways in post-stroke secondary brain injury, functional recovery, and potential therapeutic intervention can be more accurately defined.

## Data Availability

The original contributions presented in the study are included in the article/supplementary material. Further inquiries can be directed to the corresponding author.
